# Artificial intelligence across the cataract care continuum: a systematic scoping review introducing the CAICC framework and the surgical complexity prediction gap

**DOI:** 10.1186/s12886-026-05483-4

**Published:** 2026-10-27

**Authors:** Ludwig Melino Tjokrovonco, Arief S. Kartasasmita, Raden Angga Kartiwa, Rova Virgana, Chandra Prabaswara, Aisyah Nabila Azuraa

**Affiliations:** 1 Department of Ophthalmology, Faculty of Medicine, Universitas Padjadjaran, Ir. Soekarno Street km 21, Bandung, West Java 45363 Indonesia; 2 Cicendo Hospital National Eye Center, Cicendo Street No. 4, Babakan Ciamis, Bandung, West Java 40117 Indonesia; 3 Department of Ophthalmology, Universitas Padjadjaran Hospital, Cirebon–Bandung Road, Cikeruh, Sumedang, Jatinangor, West Java 45360 Indonesia; 4 Faculty of Medicine, Universitas Sebelas Maret, Ir. Sutami Street No. 36 A, Surakarta, Central Java, 57126 Indonesia

**Keywords:** Artificial intelligence, Cataract, Machine learning, Clinical validation, Clinical decision support, Surgical complexity, Intraocular lens

## Abstract

**Background:**

Cataracts remain a leading cause of reversible blindness, and artificial intelligence (AI) applications now span the cataract care pathway. However, the distribution of these applications and their validation maturity remain unclear. This review mapped AI across the care continuum, summarized task-specific performance, characterized validation maturity, and identified evidence gaps.

**Methods:**

The Preferred Reporting Items for Systematic Reviews and Meta-Analyses Extension for Scoping Reviews (PRISMA-ScR) was followed. PubMed, Scopus, the Cochrane Library, WorldCat, and the American Academy of Ophthalmology Meeting Archive were searched; the final search was completed on 20 July 2025. Eligible studies were English-language primary research published from 1 January 2020 through 20 July 2025. A review-specific cataract artificial intelligence clinical continuum (CAICC) evidence-mapping framework was developed during data charting and synthesis. Two reviewers independently conducted study selection, data extraction, endpoint adjudication, and validation-maturity assessment. Performance was summarized descriptively; validation maturity characterized evaluation independence and clinical stage, not risk of bias or overall methodological quality.

**Results:**

Of 361 reports sought for retrieval, 122 (33.8%) were not retrieved; 113/122 (92.6%) were considered plausibly eligible from available metadata. The final synthesis included 185 studies: detection (*n* = 52), characterization (*n* = 50), personalized surgical planning (*n* = 27), intelligent surgery (*n* = 28), recovery (*n* = 24), and patient engagement (*n* = 4). Among 181 validation-applicable included studies, internal testing was the most frequent highest-maturity category (123/181, 68.0%), followed by external testing (33/181, 18.2%), prospective single-center evaluation (6/181, 3.3%), and prospective multicenter evaluation (2/181, 1.1%); 17/181 (9.4%) had no independent or unclear testing. Among the retrieved and included studies, no comprehensive clinically validated multimodal preoperative cataract surgical-complexity model was identified.

**Conclusions:**

Most included studies remained at internal validation stages, and prospective multicenter evaluation was rare. Because 122/361 reports sought for retrieval were unavailable and most were considered plausibly eligible, retrieval bias may affect the comprehensiveness of the evidence map. Future research should prioritize multimodal preoperative surgical-complexity prediction, independent external testing, and prospective multicenter evaluation of locked models.

**Registration:**

Open Science Framework (OSF), DOI 10.17605/OSF.IO/ZKM86.

**Supplementary Information:**

The online version contains supplementary material available at https://doi.org/10.1186/s12886-026-05483-4.

## Background

Despite advances in cataract surgery, cataracts remain a leading cause of reversible blindness and a major public health burden, particularly in low- and middle-income countries, where population aging, increasing surgical demand, workforce shortages, and unequal access can delay treatment [1]. Cataract care involves clinical decisions across screening, diagnosis, lens characterization, biometry, intraocular lens (IOL) selection, surgical planning, intraoperative guidance, postoperative monitoring, and visual rehabilitation [2–10].

Artificial intelligence (AI) applications now extend across this pathway. Machine learning, deep learning, computer vision, foundation models, and large language models can support image interpretation, multimodal prediction, and clinical decision-making. Previous reviews have established that AI applications already span multiple stages of cataract management. Tognetto et al. systematically reviewed applications from diagnosis through follow-up and highlighted substantial heterogeneity in the available evidence [11]. Subsequent literature has expanded across cataract detection and characterization, intraocular lens (IOL) calculation, surgical-video analysis, postoperative prediction, teleophthalmology, and patient-facing applications [2–10, 12–15]. The present review therefore builds on, rather than duplicates, these earlier syntheses by providing a contemporary clinical-continuum map of the evidence, an endpoint-level audit of task-specific performance, and a structured assessment of validation maturity.

One evidence gap examined in this review lies between cataract characterization and comprehensive preoperative surgical decision support. Established cataract risk-stratification approaches can estimate defined complication risks and support counseling or case allocation [16–21]. However, these approaches primarily address specific complications or risk categories rather than a unified construct of overall operative complexity. Cataract grading algorithms likewise characterize lens opacity, subtype, or severity but do not integrate the broader determinants of operative difficulty, resource requirements, procedural modification, and surgeon-dependent risk. The gap examined in this review is therefore not the absence of cataract risk stratification, but the absence within the retrieved and included AI evidence of a comprehensive, clinically validated multimodal model for preoperative surgical-complexity assessment.

Accordingly, this systematic scoping review maps primary AI studies published from 2020 to 20 July 2025 across the cataract-care pathway using the review-specific CAICC framework, summarizes task-specific performance, characterizes validation maturity, and examines evidence gaps, with particular attention to comprehensive preoperative surgical-complexity prediction.

## Methods

### Reporting guidelines and protocol registration

This systematic scoping review was conducted and reported in accordance with the Preferred Reporting Items for Systematic Reviews and Meta-Analyses Extension for Scoping Reviews (PRISMA-ScR). The protocol was registered prospectively with the Open Science Framework (OSF; DOI: https://doi.org/10.17605/OSF.IO/ZKM86). The completed PRISMA-ScR checklist is provided in Additional file 1.

### Protocol deviations and post-registration refinements

Deviations from and refinements to the registered protocol were documented transparently. The protocol specified formal critical appraisal using the Oxford Centre for Evidence-Based Medicine framework; this appraisal was not undertaken. Instead, the completed review added a structured validation-maturity assessment focused on evaluation independence and clinical stage, which is not a risk-of-bias or overall methodological-quality assessment. The CAICC framework was developed during data charting and synthesis rather than prespecified in the registration. Endpoint-completeness and comparability adjudication and reconciled analytical task classification were also added as review-specific methodological refinements to improve traceability and prevent inappropriate combination of clinically nonequivalent outcomes. These refinements did not alter the original literature-search cutoff of 20 July 2025.

### Information sources and search strategy

A systematic search was conducted in PubMed, Scopus, and the Cochrane Library, supplemented by gray literature searches in WorldCat and the American Academy of Ophthalmology (AAO) Meeting Archive, with the final search completed on 20 July 2025. Database-specific search limits were applied as documented in Additional file 2. In PubMed, the search was run on 20 July 2025 using the database’s relative “Last 5 years” publication-date filter; on that search date, the effective publication window was approximately 20 July 2020 through 20 July 2025. The other sources used their respective year- or date-based restrictions. The search strategy combined terms related to cataracts, artificial intelligence, machine learning, deep learning, clinical prediction, image analysis, surgery, and postoperative care to capture major AI applications across the cataract care continuum.

Independently of these database-specific filters, eligibility during screening was restricted to studies published from 1 January 2020 through 20 July 2025. Thus, the relative PubMed interface filter did not exactly match the fixed screening-eligibility window. This date restriction was used to provide a contemporary mapping of a rapidly evolving evidence base, including recent developments in deep learning, clinical prediction, surgical-video analysis, and patient-facing AI applications. The lower bound should not be interpreted as the onset of AI use in cataract care; substantial foundational work predates 2020 and has been described in earlier reviews [11]. The present review was designed to characterize the recent phase of development and clinical translation rather than to provide a historical account of cataract AI. Complete source-specific search strategies are provided in Additional file 2. No search update beyond 20 July 2025 was performed as part of the present revision.

### Eligibility criteria

We included English-language primary research studies published from 1 January 2020 through 20 July 2025 that developed or independently evaluated an AI application at a clinical stage of cataract care. Independent evaluations of pre-existing AI-based formulas or systems were eligible when they tested those approaches on new clinical data. Studies were excluded if they (1) focused on conditions other than cataracts; (2) were exclusively in vitro or animal studies; (3) did not evaluate AI in a cataract patient-, eye-, imaging-, surgical-, or clinical-care context, including purely computational therapeutic-discovery studies; (4) only compared existing AI systems without a new or independently evaluated clinical AI approach; or (5) did not report primary data. Editorials, commentaries, narrative or systematic reviews, letters, and conference abstracts without accessible full-text manuscripts were excluded.

For this review, AI is operationally defined as a computational system that uses machine learning, deep learning, neural networks, or another algorithmic learning approach to perform tasks that traditionally require human cognitive processes, including pattern recognition, classification, prediction, decision support, or natural-language processing, when applied to cataract care. Eligible methods include convolutional and recurrent neural networks, transformer-based architectures, ensemble methods, support vector machines, random forests, gradient-boosting algorithms, Bayesian models, foundation models, and large language models. Systems without a learning component that performed exclusively deterministic rule-based processing were not considered AI and were excluded.

### Study selection

All records were imported into the Rayyan systematic review platform after deduplication. Two reviewers independently screened titles and abstracts. Of 1,208 screened records, 673 were excluded during the initial Rayyan screening. The 535 records retained after this stage underwent a secondary pre-retrieval eligibility verification against the prespecified disease-scope and publication-type criteria, resulting in 174 additional exclusions and leaving 361 reports sought for retrieval. Full texts were sought through publisher websites and available institutional access. When a report remained unavailable, additional searches were performed through Google Scholar, institutional or subject repositories, and ResearchGate. Reports still unavailable after these routes were classified as not retrieved and were not counted as full-text exclusions. The exact calendar date of the final full-text retrieval attempt was not prospectively recorded. Retrieved reports subsequently underwent independent full-text eligibility assessment. Disagreements were resolved through structured discussion and, when required, arbitration by a third author. Reviewer-specific pre-consensus screening decisions were not retained in a paired form that permitted retrospective calculation of raw agreement or Cohen’s kappa; accordingly, no post hoc agreement statistic was calculated from final consensus decisions. For transparency, unretrieved reports were additionally audited using the original search exports and available title and abstract metadata. Original search-source provenance was reconstructed, and eligibility plausibility was categorized as plausibly eligible, likely ineligible, or indeterminate without treating this post hoc audit as a substitute for full-text eligibility assessment.

### Data charting and extraction

Two reviewers independently charted data from the included full-text articles via a standardized Microsoft Excel workbook. The study-level variables included the first author and publication year, country of origin, study population, clinical objective, original CAICC domain, input modality, AI architecture, training and evaluation dataset characteristics, validation design, primary outcomes, and recommendations for future research. The study characteristics and original CAICC assignments are provided in Additional file 3.

For studies reporting quantitative model performance, endpoint-level data were also extracted, including the clinical task, outcome or target, metric name, reported value and unit, population or subgroup, selected model, evaluation dataset, and validation provenance. Each numerical estimate was linked to its study identifier and primary-source evidence to maintain traceability across the manuscript, additional files, and extraction workbook.

### Development and operationalization of the CAICC framework

The Cataract Artificial Intelligence Clinical Continuum (CAICC) was developed as a review-specific thematic evidence-mapping framework during data charting and synthesis. It was not used to determine study eligibility and was not intended as a validated clinical staging system, methodological-quality instrument, or clinical-readiness scale. The framework was designed to organize heterogeneous AI applications according to their principal clinical function within cataract care rather than according to algorithm type, imaging modality, or data source.

Two reviewers independently considered each study’s primary clinical objective, intended use, evaluated task, and position within the cataract-care pathway. Candidate groupings were compared and resolved by consensus into five broadly sequential clinical domains—Detection, Characterization, Personalized Surgical Planning, Intelligent Surgery, and Recovery—together with a sixth cross-cutting Patient Engagement domain. Detection included cataract identification, screening, and related case-finding tasks; Characterization included severity grading, subtype classification, lens-phenotype characterization, and related preoperative functional characterization; Personalized Surgical Planning included IOL power calculation, effective lens-position prediction, refractive prediction, and formula optimization; Intelligent Surgery included intraoperative video analysis, phase or step recognition, instrument or anatomy analysis, guidance, workflow analysis, and surgical-skill assessment; Recovery included postoperative outcome prediction, complication assessment, IOL-position prediction, posterior capsule opacification assessment, and postoperative monitoring; and Patient Engagement included patient-facing education, conversational support, telehealth, and follow-up applications.

Patient Engagement was treated as cross-cutting rather than as a strictly terminal stage because patient-facing functions may occur before, around, or after surgery. Thus, the CAICC continuum represents an evidence-mapping structure for clinical functions rather than a strictly temporal patient pathway.

The original CAICC domain denotes the study-level clinical-function assignment used for descriptive framework mapping, domain counts, and validation-maturity assessment. Following full-text endpoint adjudication, a reconciled analytical domain was added for included studies when the task actually evaluated did not correspond sufficiently to the original study-level placement. A canonical task family was then assigned to standardize clinically equivalent endpoints for task-specific performance synthesis. For studies retained in the review, these reconciled assignments did not alter study identifiers or the original six-domain CAICC counts. Eligibility adjudication was handled separately; one previously included computational therapeutic-discovery study (SUR-028) was subsequently excluded because it did not evaluate AI at a clinical stage of cataract care.

### Performance-metric completeness audit and endpoint selection

To improve the completeness and comparability of quantitative performance reporting, we conducted a targeted full-text metric-completeness audit of studies for which the initial extraction did not contain an exact numerical performance estimate. After the final eligibility adjudication, the audit comprised 77 included studies across detection (*n* = 20), characterization (*n* = 26), personalized surgical planning (*n* = 14), intelligent surgery (*n* = 11), and recovery (*n* = 6). The five-domain composition of this subset reflected the trigger for the completeness audit—absence of an exact numerical performance estimate in the initial extraction—and did not represent a separate five-domain version of CAICC. Patient Engagement remained part of the six-domain framework; its studies either already had extractable endpoint data or reported patient-facing outcomes that were not intended for conventional comparative predictive-performance synthesis.

For each audited study, the reviewers reexamined the full text and available supplementary materials to identify exact point estimates, the evaluated clinical task, the outcome definition, the metric, the unit, the study population, the model configuration, and the evaluation-set provenance. The numerical results were classified as exact performance estimates, numerical lower or upper bounds, noncomparable metrics, results without sufficient independent evaluation, or outcomes not applicable to comparative performance synthesis. Disagreements regarding endpoint interpretation, model selection, or comparability were resolved through consensus adjudication and documented in the verification log.

When multiple models or evaluation datasets were reported within a study, we preferentially selected the final proposed AI model evaluated on the most independent available dataset. For formula-comparison studies without a uniquely designated primary model, the best-performing clearly AI-based or hybrid formula was selected for each endpoint. A study contributed no more than one estimate for each comparable combination of clinical tasks, outcomes, metrics, units, and evaluation datasets.

The validation set results used for architecture selection, apparent-fit estimates from the development dataset, simulation-only outcomes, nonindependent augmented-data splits, nonselected comparator models, inequality-only results, and clinically nonequivalent surrogate outcomes were retained descriptively but excluded from the manuscript-facing performance ranges. Metrics with different definitions, including the Dice coefficient, Jaccard index, ordinary intersection over union, and frequency-weighted intersection over union, were not combined. The endpoint-level performance audit, selection decisions, and range-defining caveats are provided in Additional file 4.

### Structured validation-maturity assessment

To characterize the translational maturity of heterogeneous AI applications, we conducted a structured validation-maturity assessment of all included studies. The assessment was designed to characterize the independence and clinical stage of model evaluation rather than to generate a conventional risk-of-bias score or overall methodological-quality rating. It assessed whether evaluation was absent or unclear, internal, temporal, external, prospective single-center, or prospective multicenter.

Each study was assessed for validation applicability, study design, single-center or multicenter status, internal testing, temporal testing, external testing, prospective evaluation, and prospective multicenter evaluation. Validation applicability was classified as applicable when a study developed or evaluated a predictive, classification, segmentation, or decision-support model for which model testing was methodologically meaningful. Nonpredictive applications, such as readability transformation or primarily qualitative chatbot usability assessment, were classified as not applicable and reported separately rather than treated as inadequately validated.

Internal testing was defined as evaluation via held-out data or resampling from the same source population used for model development, including random train‒test splitting, cross-validation, bootstrap validation, or nested cross-validation. Temporal testing requires the evaluation of data collected during a later period from the same institution or source. External testing was defined as the evaluation of an independent institution, population, country, or device source not used for model development. Prospective evaluation requires assessment of a preexisting or locked model using prospectively collected patients or data, whereas prospective multicenter evaluation requires such testing at two or more clinical centers.

A mutually exclusive highest validation-maturity category was assigned to each applicable study via the following hierarchy: no independent testing or unclear testing, internal testing, temporal testing, external testing, prospective single-center evaluation, and prospective multicenter evaluation. Studies for which this taxonomy was not methodologically appropriate were retained in a separate nonapplicable category.

Before full assessment, the reviewers conducted a calibration exercise using 16 studies spanning all six CAICC domains and finalized the operational definitions in a standardized codebook. The remaining studies were independently assessed by two reviewers, with disagreements resolved through structured consensus and third-reviewer adjudication when required. Reviewer-specific pre-consensus validation-maturity classifications from the calibration and full assessment were not retained as paired decision vectors. Initial raw agreement and Cohen’s kappa could therefore not be calculated retrospectively without reconstructing reviewer decisions from the final consensus dataset, which was not considered methodologically appropriate.

The validation characteristics were summarized using frequencies and percentages. The denominator for validation applicability was the total number of studies within each CAICC domain. Percentages for internal, temporal, external, prospective, and prospective multicenter evaluations were calculated using only studies classified as validation applicable; these characteristics were not mutually exclusive. In contrast, the highest validation-maturity categories were mutually exclusive. Study-level classifications and supporting methodological evidence are provided in Additional file 5.

The validation-maturity assessment was conceptually distinct from the endpoint-selection process used for descriptive performance synthesis. Validation maturity characterized the highest stage and independence of model evaluation at the study level, whereas endpoint selection identified task-comparable numerical estimates for descriptive synthesis. The validation-maturity framework did not systematically assess other methodological domains such as participant selection, predictor measurement, reference-standard or outcome ascertainment, missing data, model overfitting, calibration, confounding, fairness, selective reporting, or reporting completeness. Accordingly, a higher validation-maturity category should not be interpreted as indicating lower risk of bias, higher overall methodological quality, or demonstrated clinical effectiveness.

### Data synthesis

The extracted data were synthesized narratively according to the CAICC clinical-function framework described above. Original CAICC domain assignments and study identifiers were retained for framework mapping, additional-file traceability, and structured validation-maturity assessment.

Following full-text endpoint adjudication, studies whose evaluated tasks did not align with their original source-domain placement were additionally assigned a reconciled analytical domain and canonical task family for manuscript-facing performance synthesis. These analytical reassignments did not alter study eligibility, original study identifiers, original six-domain counts, or source-domain validation-maturity analysis.

Quantitative performance was summarized within clinically and methodologically comparable task‒outcome‒metric groups. The exact selected point estimates were presented as observed minimum‒to‒maximum ranges together with the number of contributing estimates and unique studies. These ranges were descriptive and were not interpreted as pooled effect estimates. Metrics were not combined across different clinical tasks, outcome definitions, units, thresholds, or evaluation targets. When a special population or author-derived conversion materially influences a range boundary, the broad range is accompanied by a sensitivity analysis or an explicit interpretive caveat.

Meta-analysis was not performed because of substantial heterogeneity in clinical objectives, study populations, input modalities, AI architectures, reference standards, outcome definitions, performance metrics, and validation designs. Although the registered protocol specified formal critical appraisal using the Oxford Centre for Evidence-Based Medicine framework, that appraisal was not performed. The structured validation-maturity assessment used in the completed review characterized only the independence and clinical stage of model evaluation and was not a substitute for risk-of-bias or overall methodological-quality assessment. Consequently, observed performance ranges were treated as descriptive summaries rather than quality-adjusted, pooled, or comparative estimates. Validation characteristics were summarized using frequencies and percentages as described above.

### Use of a large language model during manuscript preparation

During manuscript preparation, the authors used ChatGPT (OpenAI) to assist with language editing, structural organization, formatting, and consistency checks of citation and extracted-data records. The tool was not used as an autonomous reviewer and did not make final decisions regarding study eligibility, data charting, endpoint selection, validation-maturity classification, or interpretation. All outputs were critically reviewed against the source material and revised by the authors, who take full responsibility for the integrity and final content of the manuscript.

## Results

**Fig. 1:**
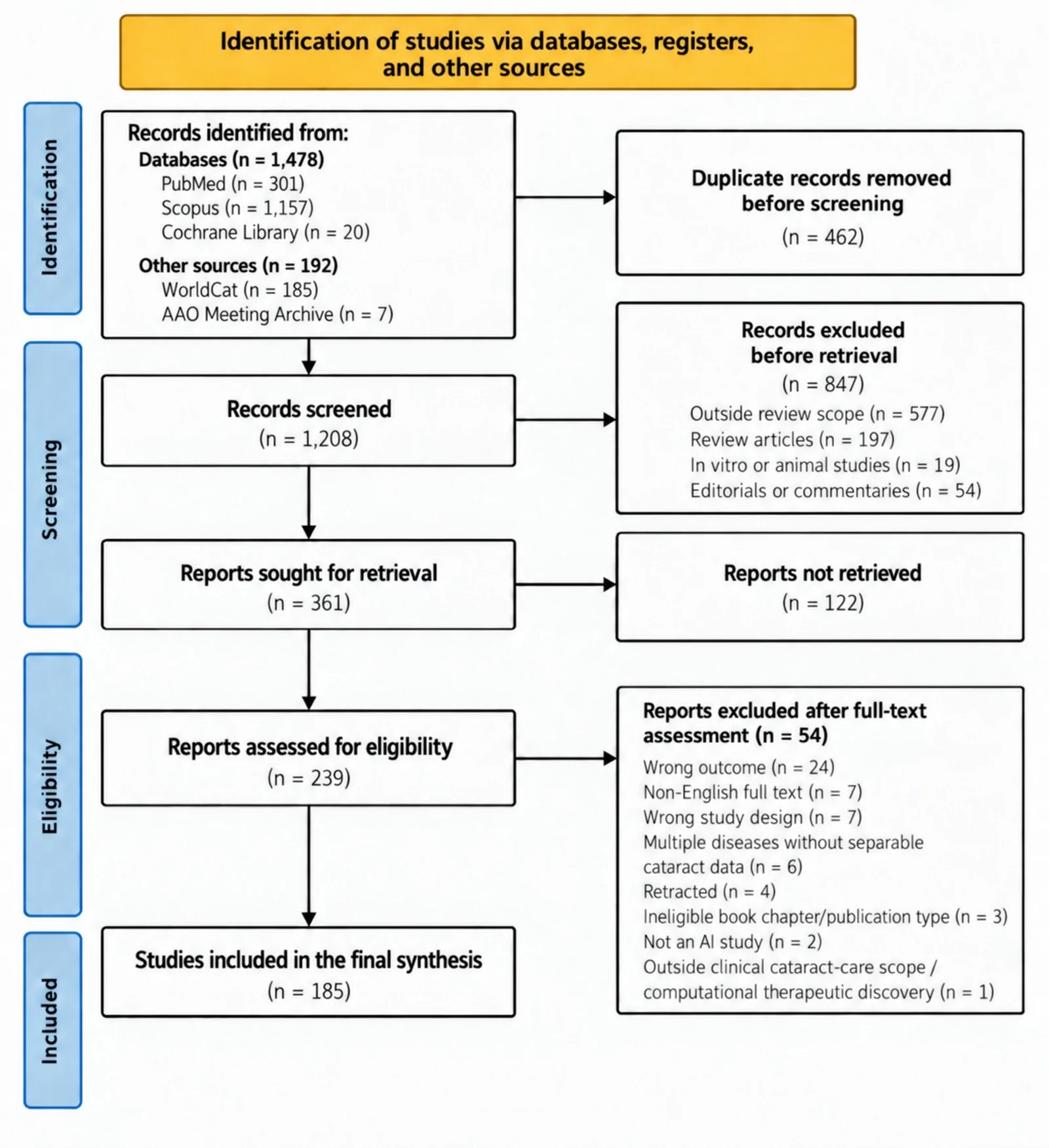
PRISMA-ScR flow diagram. The corrected flow includes 361 reports sought for retrieval, 122 reports not retrieved after the retrieval process, 239 reports assessed for eligibility, 54 full-text exclusions, and 185 included studies. Retracted reports were classified as exclusions rather than retrieval failures

The search identified 1,670 records: 1,478 from PubMed, Scopus, and the Cochrane Library and 192 from WorldCat and the American Academy of Ophthalmology Meeting Archive. After removal of 462 duplicates, 1,208 unique records underwent screening, of which 847 were excluded before full-text retrieval. Of 361 reports sought for retrieval, 122 (33.8%) remained unavailable after the retrieval process, leaving 239 reports for full-text eligibility assessment. Fifty-four reports were excluded after full-text assessment, and 185 studies were included in the final synthesis (Fig. 1). The 54 exclusions comprised wrong outcome (*n* = 24), non-English full text (*n* = 7), wrong study design (*n* = 7), multiple diseases without separable cataract data (*n* = 6), retracted reports (*n* = 4), ineligible book chapter/publication type (*n* = 3), not an AI study (*n* = 2), and one computational therapeutic-discovery study outside the clinical cataract-care scope. Individual record-level exclusion reasons were retained for 24 of the 54 full-text exclusions. For the remaining 30, the historical audit preserved exclusion status and aggregate reason counts but did not permit reliable retrospective assignment of a specific individual reason; no missing reason was reconstructed post hoc. The 122 unretrieved reports originated predominantly from Scopus (*n* = 115), with records also identified through PubMed (*n* = 16), WorldCat (*n* = 5), the AAO Meeting Archive (*n* = 3), and the Cochrane Library (*n* = 1); source counts are non-mutually exclusive because some reports were indexed in more than one source before deduplication. Their publication profile was dominated by conference and proceedings literature, while 19 reports appeared in clinical ophthalmology journals. Based on available title and abstract metadata, 113/122 (92.6%) were considered plausibly eligible, 5/122 (4.1%) likely ineligible, and 4/122 (3.3%) indeterminate.

### Overview

The 185 included studies were mapped across the following six domains of the cataract artificial intelligence clinical continuum framework: detection (*n* = 52), characterization (*n* = 50), personalized surgical planning (*n* = 27), intelligent surgery (*n* = 28), recovery (*n* = 24), and patient engagement (*n* = 4). The original CAICC domain assignments and study identifiers were retained for framework mapping, supplementary-table traceability, and structured validation-maturity assessment.

Following full-text endpoint adjudication, seven included studies received reconciled analytical assignments because their evaluated tasks did not correspond sufficiently to their original study-level CAICC placement. Six studies originally assigned to Recovery were reassigned to Characterization (REC-002, REC-016, and REC-018; *n* = 3), Detection (REC-023 and REC-024; *n* = 2), or Intelligent Surgery (REC-003; *n* = 1). One included study originally assigned to Intelligent Surgery (SUR-029) was reassigned to Cross-cutting Management because it addressed preventive drug management rather than intraoperative intelligence. The previously included computational drug-discovery study SUR-028 [22] was excluded during revision-stage eligibility adjudication because it evaluated compound screening and molecular docking without a cataract patient, eye, imaging, surgical, or clinical-care evaluation cohort. Consequently, the original Intelligent Surgery domain contained 28 included studies, the reconciled Intelligent Surgery inventory also contained 28 studies, Recovery contained 18 reconciled studies, and Cross-cutting Management contained one study. Cross-cutting Management is an analytical/narrative category and not a seventh CAICC domain. The study characteristics and original CAICC assignments are provided in Table S1 of Additional file 3; task-level quantitative performance and endpoint-selection decisions are provided in Table S2 of Additional file 4.

### Structured validation-maturity profile

Among the 185 included studies, 181 (97.8%) were applicable to the structured validation-maturity assessment. Internal testing was reported in 149/181 (82.3%), temporal testing in 2/181 (1.1%), external testing in 41/181 (22.7%), prospective evaluation of a preexisting or locked model in 8/181 (4.4%), and prospective multicenter evaluation in 2/181 (1.1%). Four included studies were not applicable to predictive-model validation: one Characterization study, one Intelligent Surgery study, and two Patient Engagement studies. The domain-specific validation characteristics and highest validation-maturity categories are summarized in Table 1.

**Table 1:** Distribution of validation characteristics and highest validation maturity across CAICC domains

Panel A. Validation characteristics							
CAICC domain	Total studies, *n*	Validation applicable, *n* (%)	Internal testing, *n* (%)	Temporal testing, *n* (%)	External testing, *n* (%)	Prospective evaluation, *n* (%)	Prospective multicenter evaluation, *n* (%)
Detection	52	52 (100.0%)	46 (88.5%)	1 (1.9%)	9 (17.3%)	3 (5.8%)	1 (1.9%)
Characterization	50	49 (98.0%)	38 (77.6%)	0 (0.0%)	4 (8.2%)	2 (4.1%)	0 (0.0%)
Personalized Surgical Planning	27	27 (100.0%)	17 (63.0%)	0 (0.0%)	15 (55.6%)	0 (0.0%)	0 (0.0%)
Intelligent Surgery	28	27 (96.4%)	24 (88.9%)	0 (0.0%)	7 (25.9%)	1 (3.7%)	0 (0.0%)
Recovery	24	24 (100.0%)	23 (95.8%)	1 (4.2%)	4 (16.7%)	1 (4.2%)	0 (0.0%)
Patient Engagement	4	2 (50.0%)	1 (50.0%)	0 (0.0%)	2 (100.0%)	1 (50.0%)	1 (50.0%)
Overall	185	181 (97.8%)	149 (82.3%)	2 (1.1%)	41 (22.7%)	8 (4.4%)	2 (1.1%)
Panel B. Highest validation maturity							
CAICC domain	No independent/unclear testing, *n* (%)	Internal testing, *n* (%)	Temporal testing, *n* (%)	External testing, *n* (%)	Prospective single-center evaluation, *n* (%)	Prospective multicenter evaluation, *n* (%)	Not applicable, *n* (%)
Detection	5 (9.6%)	38 (73.1%)	0 (0.0%)	6 (11.5%)	2 (3.8%)	1 (1.9%)	0 (0.0%)
Characterization	10 (20.4%)	35 (71.4%)	0 (0.0%)	2 (4.1%)	2 (4.1%)	0 (0.0%)	1 (2.0%)
Personalized Surgical Planning	1 (3.7%)	11 (40.7%)	0 (0.0%)	15 (55.6%)	0 (0.0%)	0 (0.0%)	0 (0.0%)
Intelligent Surgery	1 (3.7%)	19 (70.4%)	0 (0.0%)	6 (22.2%)	1 (3.7%)	0 (0.0%)	1 (3.6%)
Recovery	0 (0.0%)	20 (83.3%)	0 (0.0%)	3 (12.5%)	1 (4.2%)	0 (0.0%)	0 (0.0%)
Patient Engagement	0 (0.0%)	0 (0.0%)	0 (0.0%)	1 (50.0%)	0 (0.0%)	1 (50.0%)	2 (50.0%)
Overall	17 (9.4%)	123 (68.0%)	0 (0.0%)	33 (18.2%)	6 (3.3%)	2 (1.1%)	4 (2.2%)

Note: Values are n (%). In Panel A, validation-applicable percentages use all studies in the domain; testing-characteristic percentages use validation-applicable studies. Panel A categories are not mutually exclusive. In Panel B, highest-maturity categories are mutually exclusive; applicable-category percentages use validation-applicable studies, whereas Not applicable uses all studies in the domain. Prospective evaluation denotes evaluation of a locked model in a forward clinical cohort rather than prospective assembly of a cohort used for model development. CAICC = Cataract artificial intelligence clinical continuum. Validation maturity characterizes evaluation independence and clinical stage only and is not a risk-of-bias or overall methodological-quality score

When each applicable study was assigned a mutually exclusive highest-maturity category, internal testing was the most frequent highest-maturity category (123/181, 68.0%), followed by external testing (33/181, 18.2%), prospective single-center evaluation (6/181, 3.3%), and prospective multicenter evaluation (2/181, 1.1%); 17 studies (9.4%) had no independent testing or an unclear testing strategy. Temporal testing was identified in two studies but was not the highest-maturity category in either.

**Fig. 2:**
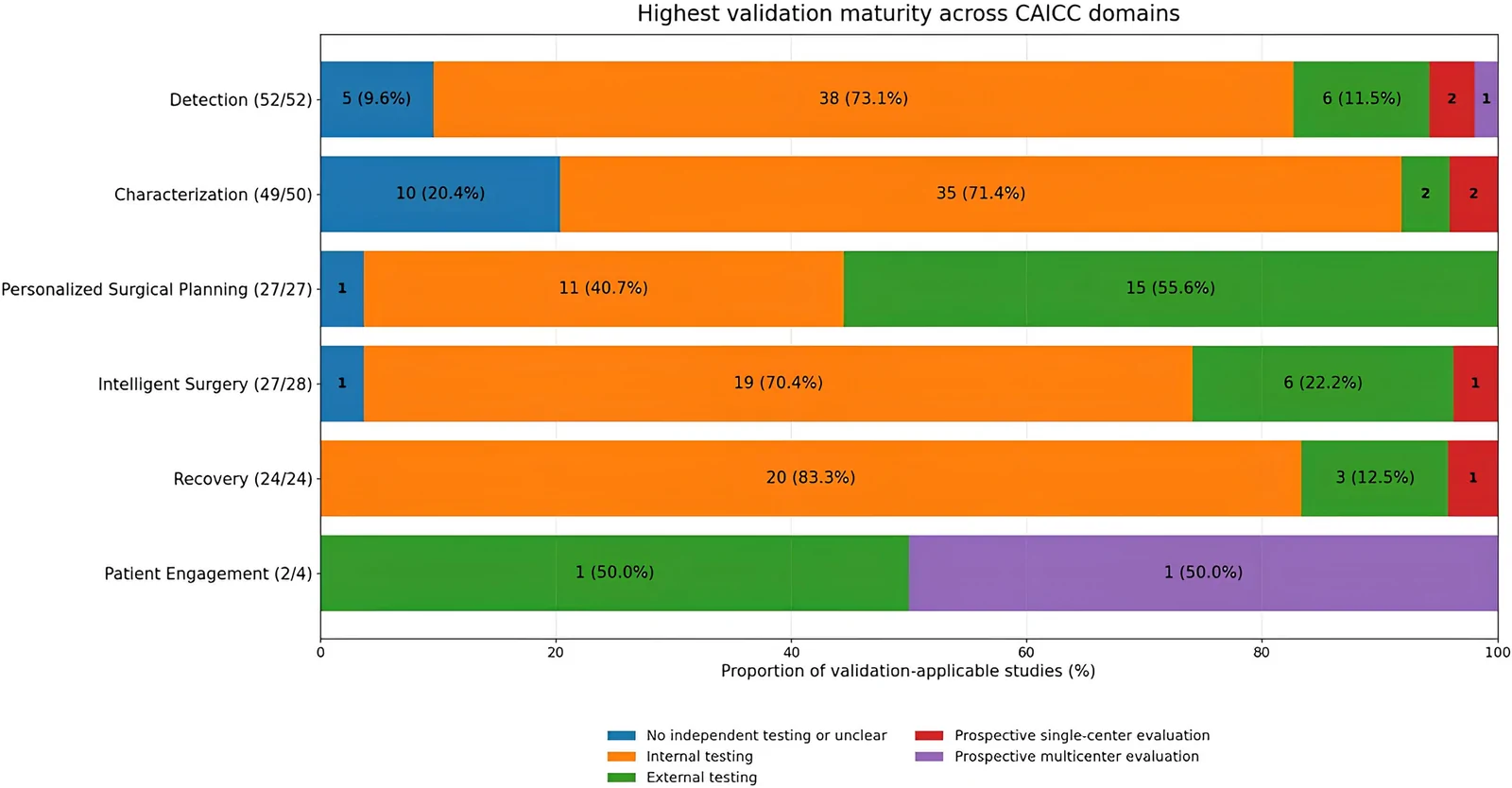
Distribution of highest validation maturity across CAICC domains. Bars show the mutually exclusive highest-maturity categories among validation-applicable studies; segment labels report study counts and percentages where space permits, while very small segments show the study count only. Labels in parentheses after each domain represent validation-applicable/total studies. Temporal testing was identified in two studies but was not the highest-maturity category in either study. CAICC = Cataract artificial intelligence clinical continuum

Validation maturity differed across CAICC domains. External testing was the most frequent highest-maturity category in Personalized Surgical Planning (15/27, 55.6%). Internal testing was the most frequent highest-maturity category in Detection (38/52, 73.1%), Characterization (35/49 validation-applicable studies, 71.4%), Intelligent Surgery (19/27 validation-applicable studies, 70.4%), and Recovery (20/24, 83.3%). These statements describe the modal highest-maturity category within each domain rather than the highest validation level reached: prospective evaluation occurred in several domains. Among the two validation-applicable Patient Engagement studies, one had external testing as its highest-maturity category and one underwent prospective multicenter evaluation. The domain-specific highest-maturity distributions are shown in Fig. 2.

### Cataract detection and characterization

#### Cataract screening and detection

Among the 52 detection studies [2, 3, 12, 23–71], deep learning architectures predominated in automated cataract identification and screening. Fundus photography was the most common input modality; slit-lamp images, anterior-segment optical coherence tomography (AS-OCT), B-scan ultrasonography, smartphone images, and clinical data were also used [2, 3, 23–27, 34, 37, 38, 41, 45, 48, 51, 54, 55, 57, 61, 67].

Recent work has increasingly addressed portable screening platforms, robustness to low-quality images, and privacy-preserving approaches such as federated learning [25, 26, 34, 37, 38, 45, 48, 51, 54, 57]. Nevertheless, most studies have relied on retrospective, single-center datasets and internal testing, whereas external or prospective evaluations remain uncommon [25, 26, 34, 37, 45, 48, 51, 57, 61, 67].

Among task-comparable cataract-detection studies, accuracy ranged from 88.50% to 96.63% across 12 selected estimates from 12 studies. area under the receiver operating characteristic curve (AUC) ranged from 0.895 to 0.997 across three estimates from three studies, sensitivity from 52.40% to 94.43% across six estimates from six studies, and specificity from 84.62% to 100.00% across six estimates from six studies. One study contributed a single eligible F1-score estimate of 90.36%. These values are descriptive observed ranges rather than pooled effects. The lower sensitivity boundary was derived from field testing of a mobile screening application, whereas the 100% specificity estimate originated from a relatively small internal test set and should therefore not be interpreted as evidence of universally perfect performance.

#### Cataract characterization and grading

A total of 50 studies [4, 13, 72–119] have investigated AI applications for cataract characterization, grading, and subtype classification. Most systems are based on convolutional neural networks trained with fundus photographs, slit-lamp images, retroillumination images, or anterior segment optical coherence tomography [4, 13, 72–77, 93, 113, 116, 119]. Common objectives include cataract severity grading according to the Lens Opacities Classification System III or related ordinal scales and the classification of nuclear, cortical, posterior subcapsular, immature, mature, or hypermature cataract phenotypes [4, 13, 72–77].

Several studies have also evaluated portable or smartphone-based platforms, near-infrared imaging, low-quality image processing, and metabolomic biomarkers for cataract characterization [76, 113, 116, 118, 119]. The reported performance varies according to the imaging modality, dataset composition, reference standard, classification task, and validation strategy, precluding direct comparisons across studies.

Among task-comparable Characterization studies, severity/state-grading accuracy ranged from 82.10% to 97.93% across nine estimates from nine studies, while subtype or morphology-classification accuracy ranged from 67.50% to 93.49% across five estimates from five studies. One additional nuclear-cataract video-classification study reported an accuracy of 94.10% and is presented as a single-study point estimate rather than combined with either grading or subtype-classification ranges. AUC values of 0.921–0.967 comprised five endpoint-specific estimates from a single study and therefore represent a within-study multi-endpoint result rather than a cross-study range. Sensitivity ranged from 88.70% to 92.80% across two studies, and specificity from 94.46% to 94.50% across two studies.

Among the included 2020–2025 AI studies, none developed and clinically evaluated a comprehensive multimodal preoperative model of overall cataract surgical complexity. Existing characterization systems primarily classified lens-opacity presence, phenotype, subtype, or severity and, in some studies, estimated associated visual functions. Studies in other CAICC domains addressed individual complexity-related components—including surgical duration, posterior capsule rupture risk, postoperative complications, pupil-expansion device use, and intraoperative workflow—but no identified AI model integrated these dimensions into a clinically validated overall preoperative complexity assessment.

### Personalized surgical planning

Twenty-seven studies investigated AI applications in personalized surgical planning, predominantly IOL power calculation and postoperative refractive prediction, including highly myopic, pediatric, postrefractive surgery, and postvitrectomy eyes [5–7, 120–143]. Across task-comparable refractive-prediction studies, mean absolute error (MAE), expressed in diopters (D), ranged from 0.279 to 0.760 D across 11 studies. Directly reported refractive median absolute error (MedAE) ranged from 0.242 to 0.680 D across nine studies. One additional study reported a median absolute IOL-plane error of 0.204 D and an author-derived Gaussian-optics conversion to a median absolute refractive error of 0.137 D; this converted value is reported separately and is not used to define the directly reported refractive MedAE range. The proportion of eyes within ± 0.50 D ranged from 39.00% to 90.38% across 12 studies.

The broader performance boundaries were influenced by clinically distinct subgroups, including pediatric eyes, and by differences in biometric platforms and evaluation designs. The 0.137 D value should therefore not be interpreted as the directly observed minimum of the refractive MedAE range. These values are descriptive selected estimates rather than pooled comparative effects and support the need for prospective external validation across diverse biometric platforms and complex subgroups before general clinical superiority can be inferred.

### Intelligent surgery

Twenty-eight included studies were originally mapped to the Intelligent Surgery domain, covering surgical phase or step recognition, instrument detection and tracking, anatomy segmentation, intraoperative guidance, surgical-skill assessment, procedure-duration prediction, and one preventive drug-management application [8, 144–170]. Following task-level reconciliation, SUR-029 [170] was analyzed narratively as Cross-cutting Management rather than intraoperative intelligence, while REC-003 was reassigned analytically from Recovery to Intelligent Surgery; the reconciled Intelligent Surgery inventory therefore remained 28 studies. The computational therapeutic-discovery study SUR-028 [22] was excluded from the final review during eligibility adjudication.

Surgical phase or step recognition was the most frequently evaluated task. Accuracy ranged from 82.64% to 98.79% across six estimates from six studies, F1 score from 77.67% to 98.79% across three estimates from three studies, precision from 82.20% to 94.90% across two estimates from two studies, recall from 77.32% to 95.20% across two estimates from two studies, and AUC from 0.950 to 0.990 across two estimates from two studies. Other task-specific findings included a pupil-segmentation Dice coefficient of 96.82% from one study, surgical-scene frequency-weighted intersection over union (FWIoU) of 90.06% from one study, surgical-skill AUCs of 0.780–0.880 across three studies, and instrument-identification AUCs of 0.970–0.999 across two studies. Different segmentation and classification metrics were reported separately and were not combined into a single performance range.

Despite generally high technical performance, most studies have relied on retrospective surgical video datasets and internal evaluations. Prospective testing of locked models across different surgeons, institutions, microscope systems, and levels of case complexity remains necessary before routine clinical implementation.

### Recovery and postoperative management

Twenty-four studies were originally mapped to the recovery domain, covering postoperative visual-outcome prediction, IOL position, depth of focus, posterior capsule opacification, cystoid macular edema, and perioperative complications [9, 171–193]. Following task-level reconciliation, 18 studies were included in the postoperative recovery synthesis, whereas six studies addressing preoperative visual-function estimation, cataract risk prediction, or intraoperative analysis were reassigned to more appropriate analytical task families.

Postoperative visual-outcome MAE ranged from 0.116 to 0.160 logarithm of the minimum angle of resolution (logMAR) across four studies. Complication-related results were not summarized as a single common range because the clinical targets differed. One study predicting broad postoperative complication risk reported an accuracy of 71.50%. A separate postoperative optical coherence tomography (OCT) study detecting retinal complications reported 92.00% accuracy, AUC 0.920, sensitivity 93.3%, and specificity 89.6%. Another study evaluating posterior capsule opacification reported external AUC 0.970, sensitivity 84.0%, and specificity 92.0%. Postoperative IOL equatorial-plane root mean square error (RMSE) ranged from 0.183 to 0.273 mm across two studies. Additional single-study findings included postoperative depth-of-focus prediction with accuracy of 70.30% and AUC of 0.802, anterior-chamber-depth MAE and MedAE of 0.105 and 0.080 mm, posterior capsule rupture area under the precision-recall curve (AUPRC) of 13.10%, and posterior capsule opacification correlation of *r* = 0.829. These are selected estimates rather than pooled effects; endpoint details are provided in Table S2 of Additional file 4.

Despite promising technical performance, most recovery studies remain limited to internal evaluation, with few externally or prospectively tested models. Differences in outcome definitions, follow-up periods, patient characteristics, and evaluation designs continue to limit direct comparison and clinical generalizability.

### Patient engagement

Four studies evaluated AI applications for patient education, communication, telehealth, and postoperative follow-up [10, 14, 15, 194]. These included readability improvement via a large language model, an expert-supervised chatbot, an AI-enabled telehealth platform for congenital cataract management, and an autonomous conversational assistant for postoperative follow-up.

Patient-engagement performance was reported by target rather than as a common cross-study range. In CC-Guardian, external endpoint-specific AUCs were 0.944 for visual-axis opacification, 0.961 for high intraocular pressure, and 0.981 for need for clinical intervention; corresponding sensitivities were 94.0%, 96.4%, and 95.9%, and specificities were 93.5%, 94.1%, and 94.5%. In the prospective multicenter Dora R1 study, the overall postoperative follow-up management decision had sensitivity of 94% and specificity of 86%. Because these systems evaluated different telehealth targets, their estimates were not combined into a single min–max range. The readability and primarily qualitative chatbot usability studies were not applicable to the predictive validation-maturity taxonomy.

### Integrated performance across the CAICC continuum

Across the CAICC continuum, reported technical performance varies substantially according to the clinical task, outcome definition, and validation design. Detection and characterization models frequently report high classification performance, whereas personalized surgical planning is predominantly evaluated via refractive-prediction errors and the proportion of eyes within predefined dioptric thresholds. Intelligent surgery studies have focused mainly on surgical workflow recognition, segmentation, instrument analysis, and skill assessment, whereas recovery studies have evaluated heterogeneous postoperative visual, anatomical, and complication-related outcomes. Patient engagement has remained the least studied and most methodologically heterogeneous domain.

The observed performance ranges summarized in Table 2 represent selected task-comparable point estimates rather than pooled effects. High technical performance did not consistently correspond to advanced validation maturity, as most models remained limited to internal testing, and relatively few underwent prospective or multicenter evaluation. Accordingly, these ranges should be interpreted as a map of reported technical performance rather than evidence of comparative clinical superiority or implementation readiness.

**Table 2:** Task-specific performance and validation profile across CAICC domains after endpoint-comparability adjudication

CAICC domain	Predominant clinical tasks	Selected observed performance (contributing estimates / unique studies)	Validation-maturity pattern	Review-identified evidence gap
Detection (*n* = 52)	Cataract screening and binary or multiclass detection	Accuracy 88.50–96.63% (12/12); AUC 0.895–0.997 (3/3); sensitivity 52.40–94.43% (6/6); specificity 84.62–100.00% (6/6)	Most frequent highest-maturity category: internal testing; several studies had no independent/unclear testing	Generalizability across populations, devices, image quality, and real-world screening environments
Characterization (*n* = 50)	Severity grading, subtype classification, and phenotype characterization	Severity/state grading accuracy 82.10–97.93% (9/9); subtype/morphology accuracy 67.50–93.49% (5/5); nuclear-cataract video classification accuracy 94.10% (1/1). AUC 0.921–0.967 (5 estimates from 1 study; within-study multi-endpoint); sensitivity 88.70–92.80% (2/2); specificity 94.46–94.50% (2/2)	Most frequent highest-maturity category: internal testing; a substantial subgroup had no independent/unclear testing	Standardized reference standards and progression from descriptive grading to clinically actionable prediction
Personalized Surgical Planning (*n* = 27)	IOL power calculation, refractive prediction, and formula optimization	MAE 0.279–0.760 D (11/11); directly reported refractive MedAE 0.242–0.680 D (9/9); author-converted refractive MedAE 0.137 D (1/1; reported separately); eyes within ± 0.50 D 39.00–90.38% (12/12)	Most frequent highest-maturity category: external testing (15/27, 55.6%)	Prospective evaluation across diverse biometric platforms and complex subgroups
Intelligent Surgery (*n* = 28)	Phase or step recognition, instrument analysis, segmentation, guidance, and skill assessment	Phase/step accuracy 82.64–98.79% (6/6); F1 77.67–98.79% (3/3); surgical-skill AUC 0.780–0.880 (3/3); pupil-segmentation Dice 96.82% (1/1; single-study point estimate)	Most frequent highest-maturity category: internal testing; evidence predominantly retrospective	Real-time multicenter evaluation across surgeons, equipment, workflows, and case complexity
Recovery (*n* = 24)	Postoperative visual prediction, IOL position, complications, and PCO assessment	Visual-outcome MAE 0.116–0.160 logMAR (4/4); broad postoperative complication-risk accuracy 71.50% (1/1); retinal-complication detection accuracy 92.00% and AUC 0.920 (1/1); PCO detection AUC 0.970 (1/1); IOL-position RMSE 0.183–0.273 mm (2/2)	Most frequent highest-maturity category: internal testing; limited external or prospective evaluation	Standardized postoperative endpoints, longitudinal follow-up, and independent prospective validation
Patient Engagement (*n* = 4)	Patient education, chatbot support, telehealth, and postoperative follow-up	CC-Guardian endpoint-specific AUCs: 0.944 (visual-axis opacification), 0.961 (high IOP), 0.981 (need for intervention), from 1 study. Dora R1 overall follow-up triage: sensitivity 94%, specificity 86% (1 study). CC-Guardian endpoint-specific sensitivity/specificity values are reported separately in Additional file 4.	Among 2 validation-applicable studies, 1 had external testing and 1 prospective multicenter evaluation as the highest-maturity category	Limited evidence base, safety, factual reliability, usability, and digital equity

Note: Observed performance values are descriptive and not pooled. A min–max range is displayed only when the clinical task, outcome target, metric, and unit are sufficiently comparable. Distinct clinical targets are presented as separate task-specific point estimates or subgroup ranges. Single-study and within-study multi-endpoint results are explicitly labelled. The 0.137 D refractive MedAE from PSP-016 is an author-derived optical conversion and is reported separately from the directly reported 0.242–0.680 D MedAE range. Patient Engagement endpoint-specific values are not combined across distinct telehealth targets. Study counts in the first column and validation profiles retain the original six-domain CAICC classification, whereas quantitative performance synthesis uses reconciled analytical task assignments after full-text endpoint adjudication. AUC = area under the receiver operating characteristic curve; CAICC = cataract artificial intelligence clinical continuum; D = diopter; IOL = intraocular lens; logMAR = logarithm of the minimum angle of resolution; MAE = mean absolute error; MedAE = median absolute error; PCO = posterior capsule opacification; RMSE = root mean square error. Detailed endpoint-selection decisions are provided in Table S2 of Additional file 4

## Discussion

### Principal findings and relation to previous evidence

This systematic scoping review identified 185 primary studies published from 2020 to July 2025 and mapped contemporary artificial intelligence applications across cataract care. Among the included studies, Detection and Characterization were the largest domains, with additional applications in Personalized Surgical Planning, Intelligent Surgery, Recovery, and a smaller cross-cutting Patient Engagement domain. Because 122 reports sought for retrieval were unavailable, these domain distributions describe the included corpus rather than the complete underlying literature. Although many included studies reported favorable technical performance, the more consistent finding was limited clinical validation. Among the 181 validation-applicable included studies, internal testing was the most frequent highest-maturity category in 123 (68.0%), external testing in 33 (18.2%), prospective single-center evaluation in 6 (3.3%), and prospective multicenter evaluation in only 2 (1.1%); 17 (9.4%) had no independent testing or an unclear testing strategy. Thus, the validation evidence within the included corpus was dominated by internal and external testing rather than prospective multicenter evaluation.

These findings extend earlier reviews rather than establishing the first synthesis of AI across cataract management. Tognetto et al. previously reviewed AI applications spanning diagnosis through follow-up and reported substantial heterogeneity that prevented quantitative synthesis [11]. The present review focuses on the more recent 2020–2025 evidence base and adds three complementary perspectives: organization according to clinical function through the CAICC framework, endpoint-level auditing of task-specific performance, and structured characterization of validation maturity. Direct numerical comparison with earlier reviews is inappropriate because eligibility criteria, search periods, and analytical methods differ, but both the earlier and present evidence indicate that heterogeneity and limited clinical validation remain important barriers to translation.

**Fig. 3:**
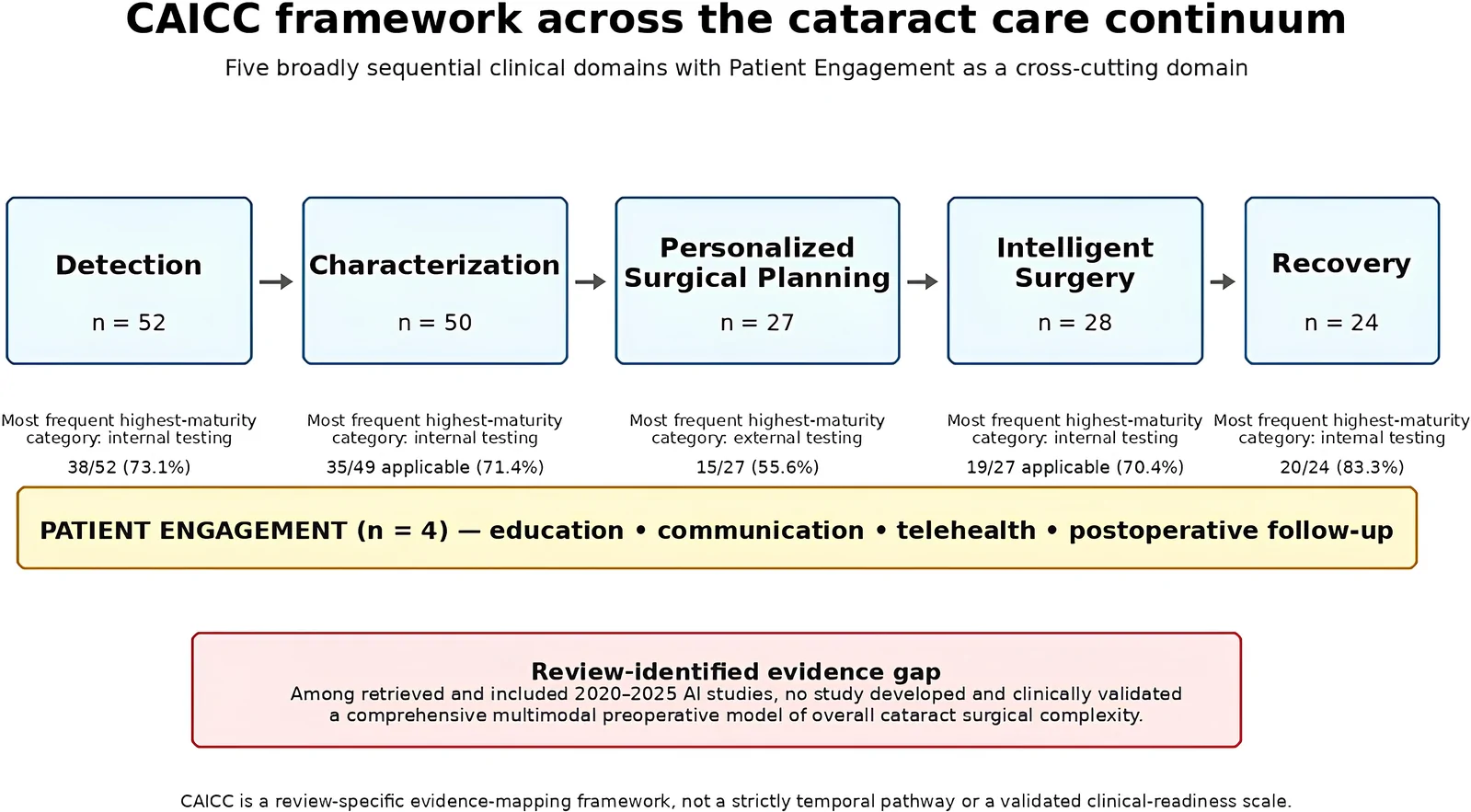
CAICC framework across the cataract care continuum. The review-specific framework organizes AI applications across five broadly sequential clinical domains—Detection, Characterization, Personalized Surgical Planning, Intelligent Surgery, and Recovery—with Patient Engagement represented as a cross-cutting domain that may operate throughout the pathway. The CAICC is an evidence-mapping framework rather than a strictly temporal clinical pathway, established clinical framework, or validated clinical-readiness scale. Domain-level validation patterns remain based on the original CAICC assignments. Within the included corpus, internal testing was the most frequent highest-maturity category in Detection, Characterization, Intelligent Surgery, and Recovery, whereas external testing was the most frequent highest-maturity category in Personalized Surgical Planning; prospective evaluation nevertheless occurred in several domains. The review-identified evidence gap was the absence, among the retrieved and included 2020–2025 AI studies, of a comprehensive clinically validated multimodal preoperative model of overall cataract surgical complexity

The CAICC framework should therefore be interpreted primarily as a review-specific evidence-mapping structure. It comprises five broadly sequential clinical domains—Detection, Characterization, Personalized Surgical Planning, Intelligent Surgery, and Recovery—with Patient Engagement operating across the pathway rather than exclusively after surgery. This organization highlights patterns and evidence gaps within the included literature without implying that the domains constitute an established or independently validated clinical staging system or a strictly temporal patient pathway. The overall CAICC framework and the review-identified evidence gap are illustrated in Fig. 3.

### Clinical and translational implications

Among the included studies, Detection and Characterization were the largest CAICC domains and contained numerous applications for automated cataract identification, grading, and phenotype analysis. However, technical discrimination or classification performance alone does not establish clinical usefulness. The observed performance ranges were derived from heterogeneous subsets of studies with differences in populations, imaging modalities, reference standards, endpoints, and evaluation designs. They should therefore be interpreted as descriptive task-specific observations rather than pooled effects or evidence that one algorithm, domain, or application is superior. Independent testing is particularly important for image-based systems because performance may change with disease prevalence, image acquisition, device characteristics, image quality, and population composition.

Personalized Surgical Planning showed the highest proportion of external testing among the five sequential CAICC domains, reflecting the established practice of comparing IOL formulas and refractive-prediction methods across independent biometric datasets. Even in this comparatively mature area, prospective evaluation of locked models remained uncommon. For Intelligent Surgery, technical advances in phase recognition, instrument analysis, segmentation, and skill assessment have created increasingly sophisticated intraoperative systems, but much of the evidence remains retrospective and dependent on limited surgical-video datasets. Recovery applications similarly encompass several potentially useful targets, including visual outcome, complications, IOL position, and posterior capsule opacification, but standardized endpoints and prospective external evaluation remain limited. Patient Engagement included promising telehealth and automated follow-up applications, including prospective evaluation, but only four studies were mapped to this domain, precluding broader conclusions.

A particularly important distinction concerns preoperative risk stratification versus overall surgical complexity. Validated cataract risk-stratification systems already exist and can estimate complication risk or support case allocation [16–21]. Conventional scoring systems have demonstrated progressively higher complication rates across increasing risk categories [16], large registry-based approaches have enabled individualized prediction of posterior capsule rupture or vitreous loss [18], and risk-based case allocation has been associated with reduced intraoperative complications in teaching settings [17]. Clinical guidance similarly recommends consideration of validated risk-stratification algorithms before cataract surgery [20]. The gap examined in this review is therefore not the absence of preoperative cataract risk assessment. Rather, among the retrieved and included 2020–2025 AI studies, we found no clinically validated multimodal model addressing the broader construct of overall operative complexity by integrating intrinsic case difficulty with information relevant to procedural planning, case allocation, resource requirements, and surgeon-dependent risk.

This distinction is clinically meaningful because surgical complexity is not synonymous with the probability of a single complication. Intrinsic factors may include lens phenotype, pupil characteristics, anterior-segment anatomy, zonular stability, previous ocular surgery, systemic or medication-related factors, and patient cooperation. Their practical consequences may be modified by the planned procedure, surgeon experience, availability of adjunctive devices, anesthesia support, and institutional resources. Future studies should therefore define the prediction target explicitly rather than using “complexity” as an unspecified composite. Clinically actionable outcomes could include posterior capsule rupture or vitreous loss, zonular dialysis, prolonged operating time, need for pupil-expansion or capsular-support devices, modification of surgical technique, senior assistance, prospectively recorded surgeon-rated difficulty, or referral to a higher-resource setting.

### Implementation priorities and future research

A key translational priority is progression from model development toward independent and prospective evaluation of locked systems. External validation should evaluate clinically relevant distribution shifts, including different institutions, populations, devices, surgeons, and workflows. Prospective studies should assess not only technical discrimination but also calibration, failure modes, usability, clinical decision consequences, workflow integration, and patient outcomes. Multicenter evaluation is particularly important for systems intended to influence surgical planning or intraoperative decisions.

Implementation also requires attention to transparency, governance, and human oversight [195, 196]. Inadequate reporting of dataset provenance, recruitment, model locking, data partitioning, and evaluation independence complicates interpretation and reproducibility. Systems affecting patient counseling, surgical planning, or intraoperative management require clearly defined intended use and responsibility for final clinical decisions. Postdeployment monitoring will also be necessary because performance may deteriorate when models encounter new populations, equipment, clinical practices, or changing data distributions.

Resource requirements should be considered during model development rather than after technical validation [197, 198]. Systems developed around advanced imaging, integrated electronic health records, and high-resource operating environments may not transfer directly to settings where these infrastructures are unavailable. Resource-adapted pathways may instead require smartphone or handheld imaging, lower-cost biometry, structured clinical assessment, intermittent connectivity, and simplified deployment. Importantly, these should not be treated merely as reduced versions of tertiary-center systems; they require independent development and evaluation for local feasibility, affordability, maintenance, image quality, usability, referral pathways, and digital equity.

Future cataract-AI research would therefore benefit from fewer incremental demonstrations on isolated internal datasets and greater emphasis on clinically defined problems. Priorities include prospective validation of established applications, multimodal preoperative decision support, reproducibility across equipment and populations, patient-facing safety and factual reliability, and evaluation of whether AI changes clinical decisions or outcomes. A hypothesis-generating framework of candidate variables, contextual modifiers, prediction targets, and resource-adapted data sources for future surgical-complexity model development is provided in Additional file 7; it is a research agenda rather than a clinical prediction score.

### Strengths and limitations

This review has several strengths. It provides a contemporary mapping of a large and heterogeneous cataract-AI literature according to clinical function rather than algorithm type alone. Prospective protocol registration, PRISMA-ScR reporting, independent study selection and data charting, and standardized endpoint adjudication improved methodological transparency. The endpoint-level performance audit linked selected estimates to their clinical task, model, evaluation dataset, and source evidence, while the structured validation-maturity assessment distinguished the presence of testing procedures from the highest level of evaluation achieved. These approaches allowed technical performance and translational maturity to be considered separately.

Several limitations require emphasis. First, the search included PubMed, Scopus, the Cochrane Library, WorldCat, and the American Academy of Ophthalmology Meeting Archive but did not include IEEE Xplore, Embase, or Web of Science; relevant engineering or multidisciplinary studies may therefore have been missed. This limitation is particularly relevant to engineering-oriented AI research and IEEE-indexed conference literature. The review was also restricted to English-language studies published from January 2020 onward. In addition, PubMed was searched with the relative “Last 5 years” filter on 20 July 2025, corresponding to an effective lower boundary of approximately 20 July 2020, rather than the fixed 1 January 2020 eligibility boundary used during screening. Other included sources used 2020-inclusive year or date restrictions, but the PubMed window should not be interpreted as fully covering January through 19 July 2020. The overall date restriction was intended to provide a contemporary evidence map and should not be interpreted as the beginning of AI research in cataract care; foundational earlier studies are outside the scope of the review.

Second, 122 of 361 reports sought for retrieval (33.8%) remained unavailable after attempts through publisher websites, institutional access, Google Scholar, repositories, and ResearchGate. The missing set was dominated by conference and proceedings literature, but 19 unretrieved reports appeared in clinical ophthalmology journals. Based on available title and abstract information, 113/122 (92.6%) were considered plausibly eligible. The unretrieved material therefore may not represent a random subset of the literature, and retrieval bias could have affected both the comprehensiveness of the evidence map and the apparent distribution of applications across CAICC domains. Domain-level counts and absence statements in this review should consequently be interpreted as applying to the retrieved and included corpus. The exclusion of conference abstracts without accessible full-text reports may have further reduced representation of early-stage engineering research.

Third, although the registered protocol specified formal critical appraisal using the Oxford Centre for Evidence-Based Medicine framework, no formal risk-of-bias or overall methodological-quality assessment was completed. This limits interpretation of the reported technical performance of included AI models. The validation-maturity framework assessed a narrower methodological dimension: the independence and clinical stage of model evaluation. It did not comprehensively evaluate participant selection, predictor measurement, outcome ascertainment, missing data, overfitting, calibration, confounding, fairness, selective reporting, or reporting quality. Validation maturity, reported model performance, and overall methodological quality should therefore be interpreted as distinct dimensions, and a study classified at a higher validation-maturity level should not automatically be interpreted as having low risk of bias or demonstrated clinical effectiveness.

Fourth, validation and performance classifications depended on methodological information available in the primary reports. Incomplete descriptions of institutional provenance, recruitment direction, dataset splitting, model locking, or evaluation independence may have resulted in conservative or uncertain classifications. “External validation” also encompassed different forms of distribution shift—including institution, population, country, and device—which are not methodologically equivalent.

Finally, substantial heterogeneity in clinical objectives, populations, data sources, reference standards, AI architectures, endpoints, and validation designs prevented meta-analysis and limits direct comparisons between applications. Reviewer-specific pre-consensus screening and validation-maturity decisions were not retained in a paired form, preventing retrospective calculation of initial agreement statistics. The proposed future surgical-complexity research agenda was informed by the evidence gap identified in this review and established cataract risk literature but was not empirically derived, statistically weighted, or developed through a formal consensus process. It should not be used for individual clinical decision-making without subsequent model development and prospective validation.

## Conclusions

This systematic scoping review mapped 185 included studies of AI across the cataract care continuum. Within the included validation-applicable corpus, internal testing was the most frequent highest-maturity category in 123/181 studies (68.0%), whereas only 2/181 (1.1%) reached prospective multicenter evaluation; 17/181 (9.4%) had no independent testing or an unclear testing strategy. High technical performance should therefore not be equated with generalizability, clinical utility, overall methodological quality, or readiness for implementation. These findings should also be interpreted in light of the substantial and potentially non-random non-retrieval of 122/361 reports sought for retrieval.

The review-specific CAICC framework highlights an evidence gap within the retrieved and included literature: among the 2020–2025 AI studies in the final synthesis, no study developed and clinically validated a comprehensive multimodal preoperative model of overall cataract surgical complexity. Future models should integrate patient- and case-level determinants—including lens phenotype, ocular anatomy, medication-related factors, and patient cooperation—with contextual modifiers such as the planned procedure, surgeon experience, and available institutional resources. Such models should define clinically actionable outcomes, undergo independent external validation, and be prospectively evaluated across centers before use for case allocation, operative preparation, or individualized risk management.

## Supplementary Information

Below is the link to the electronic supplementary material.

Supplementary Material 1

Supplementary Material 2

Supplementary Material 3

Supplementary Material 4

Supplementary Material 5

Supplementary Material 6

Supplementary Material 7

## Data Availability

All data generated or analyzed during this study are included in this article and its additional files. The complete search strategies, study-level characteristics, endpoint-level performance data and selection decisions, and structured validation-maturity assessments are provided in Additional files 2–5.

## Abbreviations

AAO: American academy of ophthalmology
AI: Artificial intelligence
AS-OCT: Anterior-segment optical coherence tomography
AUC: Area under the receiver operating characteristic curve
AUPRC: Area under the precision-recall curve
CAICC: Cataract artificial intelligence clinical continuum
D: Diopter
FWIoU: Frequency-weighted intersection over union
IOL: Intraocular lens
logMAR: logarithm of the minimum angle of resolution
MAE: Mean absolute error
MedAE: Median absolute error
OCT: Optical coherence tomography
OSF: Open science framework
PCO: Posterior capsule opacification
PRISMA-ScR: Preferred reporting items for systematic reviews and meta-analyses extension for scoping reviews
RMSE: Root mean square error
