# Supplementary material for: Artificial intelligence across the cataract care continuum: a systematic scoping review introducing the CAICC framework and the surgical complexity prediction gap
Source: BMC Ophthalmol. 2026 Oct 27;26:696. doi: 10.1186/s12886-026-05483-4 (PMC13718037; doi:10.1186/s12886-026-05483-4)
Supplement: Supplementary file 1 [file 12886_2026_5483_MOESM1_ESM.docx]

**Additional file 1. PRISMA-ScR reporting checklist**

Review title: Artificial intelligence across the cataract care continuum: a systematic scoping review introducing the CAICC framework and the surgical complexity prediction gap

**Guideline:** PRISMA Extension for Scoping Reviews (PRISMA-ScR). Tricco AC, Lillie E, Zarin W, et al. Ann Intern Med. 2018;169(7):467–473. doi:10.7326/M18-0850.

| **Section / item** | **Reporting requirement (paraphrased)** | **Location in manuscript** | **Status** |
| --- | --- | --- | --- |
| TITLE 1 | Identify the report as a scoping review. | Title page, p. 1 | Reported |
| ABSTRACT 2 | Structured summary of rationale, objectives, eligibility, sources, charting, results, and conclusions. | Abstract, p. 2 | Reported |
| INTRODUCTION 3 | Describe the rationale in the context of existing knowledge. | Background, pp. 3–4 | Reported |
| INTRODUCTION 4 | State review questions/objectives, concepts, and scope. | Background, p. 4 | Reported |
| METHODS 5 | State protocol availability and registration. | Methods, p. 4 | Reported |
| METHODS 6 | Specify eligibility criteria and their application. | Methods, pp. 5–6 | Reported |
| METHODS 7 | Describe information sources and the final search date. | Methods, p. 5 | Reported |
| METHODS 8 | Present a complete electronic search strategy for at least one source. | Additional file 2 | Reported |
| METHODS 9 | Describe selection of sources of evidence. | Methods, p. 6 | Reported |
| METHODS 10 | Describe data charting, reviewer independence/calibration, and disagreement resolution. | Methods, pp. 6–10 | Reported |
| METHODS 11 | List and define data items sought. | Methods, pp. 7–10 | Reported |
| METHODS 12 | When performed, explain critical appraisal and its use in synthesis. | Methods, pp. 4, 9–12; Strengths and limitations, pp. 30–32 | Not performed (optional) |
| METHODS 13 | Describe handling and synthesis of charted data. | Methods, pp. 8–11 | Reported |
| RESULTS 14 | Report study selection and exclusions, preferably with a flow diagram. | Results, pp. 12–13; Figure 1; Additional file 6 | Reported |
| RESULTS 15 | Present characteristics of included sources and cite them. | Results, pp. 13–15; Additional file 3 | Reported |
| RESULTS 16 | When performed, present critical-appraisal results. | Not applicable | Not performed (optional) |
| RESULTS 17 | Present relevant charted data for individual sources. | Additional files 3–5 | Reported |
| RESULTS 18 | Summarize charting results in relation to the review objectives. | Results, pp. 12–25; Tables 1–2; Figure 2 (p. 17); Figure 3 (p. 27); Additional files 3–5 | Reported |
| DISCUSSION 19 | Summarize the main findings, concepts, and evidence types. | Discussion, pp. 26–32 | Reported |
| DISCUSSION 20 | Discuss limitations of the scoping-review process. | Strengths and limitations, pp. 30–32 | Reported |
| DISCUSSION 21 | Provide an overall interpretation and implications/next steps. | Discussion, pp. 26–32; Conclusions, p. 32 | Reported |
| FUNDING 22 | Describe funding for included evidence and for the review. | Declarations: Funding, p. 34 | Partly reported* |

Notes: Items 12 and 16 are optional. Formal critical appraisal specified in the registered protocol was not performed; the structured validation-maturity assessment was used instead to characterize evaluation independence and clinical stage and was not treated as a formal risk-of-bias or overall methodological-quality appraisal. Page references are synchronized to the final 185-study submission-ready minor-revision manuscript. *Review funding is reported, but funding of each included study was not charted.
