# Supplementary material for: Artificial intelligence across the cataract care continuum: a systematic scoping review introducing the CAICC framework and the surgical complexity prediction gap
Source: BMC Ophthalmol. 2026 Oct 27;26:696. doi: 10.1186/s12886-026-05483-4 (PMC13718037; doi:10.1186/s12886-026-05483-4)
Supplement: Supplementary file 2 [file 12886_2026_5483_MOESM2_ESM.docx]

**Additional file 2. Complete search strategies**

**Final search cutoff: 20 July 2025**

Search dates and database-specific limits. Searches used source-specific interfaces and limits, with the final search completed on 20 July 2025. PubMed was run that day using the relative "Last 5 years" publication-date filter, corresponding to an effective window of approximately 20 July 2020 through 20 July 2025. Other sources used the year- or date-based restrictions shown below. Screening eligibility was independently fixed at 1 January 2020 through 20 July 2025; therefore, PubMed did not fully cover 1 January through 19 July 2020. All filters are reported as originally applied and were not retrospectively replaced. No search update beyond 20 July 2025 was performed.

| **Source** | **Records** | **Original database-specific filters/limits and cutoff application** | **Exact original search strategy** |
| --- | --- | --- | --- |
| PubMed | 301 | Original PubMed "Last 5 years" filter (Publication Date), run on 20 July 2025; effective publication window approximately 20 July 2020 through 20 July 2025. Reported as originally applied and not replaced by a fixed date query. Screening eligibility was independently fixed at 1 January 2020 through 20 July 2025, so PubMed did not fully cover 1 January through 19 July 2020. | (((cataract[MeSH Terms]) OR (cataract[Title/Abstract])) AND (((((((artificial intelligence[MeSH Terms]) OR (artificial intelligence[Title/Abstract])) OR (AI[Title/Abstract])) OR (machine learning[Title/Abstract])) OR (deep neural network[Title/Abstract])) OR (convolutional neural network[Title/Abstract])) OR (deep learning[Title/Abstract]))) AND ((((((((diagnos*[Title/Abstract]) OR (surgery[Title/Abstract])) OR (intraocular lens[Title/Abstract])) OR (prognosis[Title/Abstract])) OR (visual acuity[Title/Abstract])) OR (detection[Title/Abstract])) OR (risk[Title/Abstract])) OR (complication[Title/Abstract])) |
| Scopus | 1,157 | Publication years 2020-2025 (PUBYEAR > 2019 AND PUBYEAR < 2026). During screening, eligibility was restricted to publications from 1 January 2020 through 20 July 2025. | ( TITLE-ABS-KEY ( cataract ) ) AND ( ( TITLE-ABS-KEY ( artificial AND intelligence ) ) OR ( TITLE-ABS-KEY ( ai ) ) OR ( TITLE-ABS-KEY ( machine AND learning ) ) OR ( TITLE-ABS-KEY ( deep AND neural AND network ) ) OR ( TITLE-ABS-KEY ( deep AND learning ) ) OR ( TITLE-ABS-KEY ( convolutional AND neural AND network ) ) ) AND ( ( TITLE-ABS-KEY ( diagnosis ) ) OR ( TITLE-ABS-KEY ( surgery ) ) OR ( TITLE-ABS-KEY ( intraocular lens ) ) OR ( TITLE-ABS-KEY ( prognosis ) ) OR ( TITLE-ABS-KEY ( visual acuity ) ) OR ( TITLE-ABS-KEY ( detection ) ) OR ( TITLE-ABS-KEY ( risk ) ) OR ( TITLE-ABS-KEY ( complication ) ) ) AND PUBYEAR > 2019 AND PUBYEAR < 2026 |
| Cochrane Library | 20 | Reviews, Protocols, and Trials; original custom date range: 1 January 2020 to 20 July 2025. | #1 cataract #2 artificial intelligence #3 AI #4 machine learning #5 deep neural network #6 convolutional neural network #7 deep learning #8 diagnos* #9 surgery #10 intraocular lens #11 prognosis #12 visual acuity #13 detection #14 risk #15 complication #16 #2 OR #3 OR #4 OR #5 OR #6 OR #7 #17 #8 OR #9 OR #10 OR #11 OR #12 OR #13 OR #14 OR #15 #18 #1 AND #16 AND #17 Original date limit applied in the Cochrane interface: 1 January 2020 to 20 July 2025. |
| WorldCat | 185 | Formats: thesis, dissertation, article, downloadable article, and downloadable archival material; Language: English; Year: 2020-2025. During screening, eligibility was restricted to publications from 1 January 2020 through 20 July 2025. | Format: Thesis, dissertation, article, downloadable article, downloadable archival material Language: English Year: 2020–2025  kw:cataract AND kw:artificial intelligence AND kw:(diagnosis OR surgery OR intraocular lens OR prognosis OR visual acuity OR detection OR risk OR complication) Screening eligibility cutoff: 20 July 2025. |
| AAO Meeting Archive | 7 | Keyword: artificial intelligence; Topic: cataract; annual searches for 2020-2025. During screening, eligibility was restricted to material published through 20 July 2025. | Keyword: artificial intelligence Topic: cataract Search repeated separately for each year from 2020 through 2025. Screening eligibility cutoff: 20 July 2025. |

Record-count reconciliation: PubMed, Scopus, and the Cochrane Library contributed 1,478 records; WorldCat and the AAO Meeting Archive contributed 192 records; total identified records = 1,670.

Table note: PubMed's relative "Last 5 years" filter, run on 20 July 2025, corresponded to an effective publication window of approximately 20 July 2020 to 20 July 2025. The fixed 1 January 2020 to 20 July 2025 period refers to screening eligibility. No updated search beyond 20 July 2025 was performed.
