# Supplementary material for: Artificial intelligence across the cataract care continuum: a systematic scoping review introducing the CAICC framework and the surgical complexity prediction gap
Source: BMC Ophthalmol. 2026 Oct 27;26:696. doi: 10.1186/s12886-026-05483-4 (PMC13718037; doi:10.1186/s12886-026-05483-4)
Supplement: Supplementary file 3 [file 12886_2026_5483_MOESM7_ESM.docx]

Additional file 7. Conceptual research agenda for multimodal cataract surgical-complexity models

Purpose and status. This document is a hypothesis-generating research agenda informed by the evidence gap identified in the scoping review and by established cataract risk-stratification and complication literature (see manuscript references 16–21). It was not derived from a formal consensus process, predictive-model development procedure, statistical feature-selection analysis, or prospective validation study. It is not a clinical score and should not be used for patient-level decision-making without subsequent empirical model development and validation.

**Conceptual distinction.** Future work should distinguish intrinsic case complexity from context-dependent operative risk. Intrinsic patient and ocular factors may have different clinical consequences depending on the planned procedure, surgeon experience, available adjunctive devices, anesthesia support, and institutional resources.

# Table S5A. Candidate intrinsic case factors and contextual modifiers

| **Layer** | **Candidate construct** | **Illustrative variables** | **Preferred data sources** | **Resource-adapted alternatives** | **Intended role in future modeling** |
| --- | --- | --- | --- | --- | --- |
| Intrinsic case factors | Ocular structural and anatomical features | Anterior-chamber depth and angle configuration; axial length; pupil diameter and dilatability; corneal clarity; endothelial-cell reserve; previous ocular surgery; pseudoexfoliation, phacodonesis, lens subluxation, or other signs of zonular weakness | AS-OCT; optical biometry; specular microscopy; slit-lamp examination; electronic health records | Ultrasound biometry; handheld slit lamp; smartphone anterior-segment imaging; torchlight pupil assessment; structured clinical checklist | Estimate operative working space, visualization, capsular/zonular stability, endothelial vulnerability, and likely need for adjunctive devices. |
| Intrinsic case factors | Lens phenotype | Nuclear density or hardness; brunescent, white, intumescent, mature, or hypermature cataract; posterior polar cataract; cortical or posterior subcapsular components; lens position and stability | Standardized slit-lamp photography; Scheimpflug imaging; AS-OCT; objective lens-density analysis | Standardized smartphone or handheld slit-lamp photography with controlled illumination and clinician grading | Anticipate phacoemulsification energy, capsulorhexis difficulty, posterior-capsule vulnerability, and possible modification of surgical technique. |
| Intrinsic case factors | Systemic and medication-related factors | Alpha-1 antagonist exposure and IFIS risk; tremor or movement disorder; respiratory or musculoskeletal limitations affecting positioning; relevant systemic comorbidities | Electronic health records; medication reconciliation; structured medical assessment | Paper or mobile preoperative questionnaire; medication checklist | Identify factors affecting pupil behavior, positioning, anesthesia requirements, and intraoperative stability. |
| Intrinsic case factors | Behavioral and cooperation factors | Ability to lie flat; fixation and cooperation; cognitive impairment; anxiety; hearing or communication difficulty; involuntary movements | Preoperative clinical assessment; standardized cooperation questionnaire; anesthesia assessment | Brief structured interview; direct observation | Support selection of anesthesia, communication strategy, positioning, and need for additional assistance. |
| Contextual modifiers | Surgeon and procedural context | Surgeon experience or competency level; trainee involvement; planned technique; type of anesthesia; availability of senior supervision; anticipated need for trypan blue, pupil-expansion devices, capsular-tension devices, vitrectomy equipment, or alternative extraction techniques | Surgical roster; training records; operating-theatre information system; preoperative surgical plan | Local surgeon-competency categories; facility-resource checklist | Translate intrinsic case difficulty into context-specific operative risk and support case allocation, equipment preparation, and senior-assistance planning. |
| Contextual modifiers | Health-system and resource context | Imaging availability; equipment capability; surgical volume; referral distance; availability of vitreoretinal or anesthesia support; capacity for postoperative follow-up | Institutional records; theatre-resource data | Facility-level checklist | Determine whether a case can be managed locally or should be referred to a higher-resource setting. |

# Table S5B. Candidate clinically actionable prediction targets

| **Outcome class** | **Examples** | **Potential clinical use** |
| --- | --- | --- |
| Specific intraoperative complications | Posterior capsule rupture or vitreous loss; zonular dialysis; iris complications | Risk counseling; surgeon allocation; contingency planning; senior supervision. |
| Technical difficulty / procedural modification | Prolonged operating time; need for pupil-expansion or capsular-support devices; conversion or modification of surgical technique | Equipment preparation; theatre scheduling; procedure planning. |
| Human-resource requirements | Need for senior assistance; need for anesthesia support; surgeon-rated difficulty recorded prospectively | Case allocation; training supervision; staffing. |
| Site-of-care decision | Referral or escalation to a higher-resource surgical setting | Regional triage; referral planning; resource-sensitive implementation. |

**Recommended development principles.** Prespecify the target outcome; separate intrinsic case complexity from contextual modifiers; evaluate incremental value beyond established risk stratification; assess calibration and clinically relevant failure modes; externally validate across institutions, devices, surgeons, and populations; prospectively evaluate locked models; and develop resource-adapted pathways as independent implementation strategies rather than assuming transferability from tertiary-center systems.
